# Microsurgical treatment of ophthalmic artery aneurysm, a case series of 55 patients with long-term follow-up

**DOI:** 10.1186/s12893-024-02419-x

**Published:** 2024-05-07

**Authors:** Abdolkarim Rahmanian, Ehsan Mohammad Hosseini, Arman Sourani, Mohammad Jamali, Arash Saffarian, Keyvan Eghbal, Sanaz Taherpour, Mina Foroughi

**Affiliations:** 1 Neurosurgery department, Shiraz University of Medical Sciences, Shiraz, Iran; 2 Department of Neurosurgery, Isfahan University of Medical Sciences, Isfahan, Iran; 3 Isfahan Students’ Research Committee (ISRC), Isfahan University of Medical Sciences, Isfahan, Iran; 4 Environment Research Center, Research Institute for Primordial Prevention of Non-Communicable Disease, Isfahan University of Medical Sciences, Isfahan, Iran

**Keywords:** Ophthalmic artery aneurysm, Paraclinoidal artery aneurysm, Aneurysm microsurgery, Lateral supra orbital craniotomy, Brain aneurysm, Clip ligation

## Abstract

**Background:**

Ophthalmic artery aneurysm (OAA) can be secured in endovascular or microsurgical approaches. Still there are controversies in technique selection and their long term outcomes.

**Methods:**

All the patients with OAA were treated microsurgically and followed. Demographic data, neurological status, physical examination findings, angiographic data, operation details, and intraoperative and postoperative events were recorded and analyzed. *P* < 0.05 was considered significant.

**Results:**

Among 55 patients, 38 were females (69.1%). Median preoperative glasgow coma scale (GCS), Fisher Grade, and Hunt and Hess(HH) scores were 15, 1 and 1, respectively. The most common neurologic manifestation was visual problems (*n* = 15). The most common anatomical projection was medial (43.6%) oriented lesions. 85.5% of them only had 1 ophthalmic aneurysm while multiple aneurysms were reported in 14.6%. In 52 patients temporary clip was used. in 21 patients (38.2%) intraoperative aneurysm rupture occurred. Larger aneurysm size and preoperative hydrocephalus were associated with higher rates of aneurysm rupture (*P* = 0.003 and 0.031). 28.5% of the patients with visual problems had clinical improvement in the postoperative period. The mean follow-up period was 5 years. Follow-up angiography showed a 100% obliteration rate with a 0.0% recurrence rate. Median values for follow-up glasgow outcome scale and modified Rankin scale were 5 and 0, respectively. favorable neurological outcomes were associated with better primary GCS and HH scores.

**Conclusion:**

OAA microsurgery is an effective and safe procedure with significant improvement in both visual and neurological status. Low recurrence rate and excellent clinical recovery are the most important advantages of microsurgery in OAA treatment.

**Supplementary Information:**

The online version contains supplementary material available at 10.1186/s12893-024-02419-x.

## Introduction

Ophthalmic artery aneurysms (OAA) are rare intracranial aneurysms that arise from the ophthalmic or paraclinoidal segment of the internal carotid artery. In most cases, it gives rise from ICA adjacent to the optic nerve (ON) and is presented with visual manifestations. Visual loss and visual field defects are the most common ophthalmologic presentations of OAA while SAH, ICH, hydrocephalus, and cranial nerve deficits are other clinical presentations of OAA. There are two main approaches for aneurysm occlusion, endovascular and microsurgical techniques [1].

In endovascular (EV) approaches, via arterial access neuro-interventionist passes the guide catheters to the targeted vessels and confirms the location of the aneurysm with intraoperative angiographic fluoroscopy, then occludes the lesions using multiple agents or devices. EV is less invasive in comparison to surgical approaches, and it is effective in narrow-necked aneurysms but it has a greater risk of treatment failure and/or recurrence in comparison to microsurgery [2].

In the microsurgical approach, following a craniotomy and considering the approach, arachnoid dissection is performed, optic apparatus and lamina terminalis is dissected, and the ophthalmic segment of ICA is skeletonized. Then, using titanium clips, the aneurysm will be secured and after proper hemostasis the flap is refixed. In this technique, the neurosurgeon usually exposes the proximal segment of the feeding artery depending on the location of the aneurysm [3]. This technique is an essential step in microsurgical approaches for intracranial aneurysms providing proximal control on the feeding artery in cases of aneurysm rupture or providing better exploration of the surgical field [1].

In general, microsurgery is used for medium-sized vessels, especially in distal arterioles which EV access is technically challenging. Hematoma evacuation, lower risk of treatment failure, lower recurrence rate, and better bleeding control are important advantages of microsurgical approaches in comparison to EV for aneurysm obliteration [4]. In anatomical complex regions such as paraclinoidal segment aneurysms, tortuous and complex vascular anatomy makes the EV challenging while MS provides great anatomical visualization, safe vascular exposure, and effective postoperative outcomes. Compared to endovascular techniques, direct decompression of neural structure following the microsurgical approaches, is extremely beneficial for neurological recovery [4].

There is no consensus on the best treatment modality for paraclinoidal aneurysms but due to anatomical complexity, greater risk of bleeding, and mass effect of hematoma/aneurysm on oculomotor and optic nerves, microsurgical approaches seem to be a more curative treatment option [3].

In this clinical study, we have treated 55 cases of ophthalmic artery aneurysm in a microsurgical approach and followed them for a long-term period.

## Methods

### Study design and the participants

This study is a prospective non-randomized case series that was held in Namazi Neurosurgical Center, Shiraz, Iran from 2010 to 2022. Namazi Hospital is a high-volume center for all neurosurgical disciplines. Specialized hybrid endovascular-microvascular neurosurgeons are treating vascular lesions regularly, and the annual aneurysm microsurgery rate exceeds 100–120 procedures in Namazi Hospital.

This study was approved and supervised by the Shiraz University of Medical Sciences Ethics Committee board. Informed medical consent was obtained from all the patients and/or their relatives for medical treatments and research purposes.

All the patients who were referred or diagnosed with ophthalmic artery aneurysm (OAA) were enrolled in the study. This included both ruptured and unruptured OAAs.

In terms of timing from diagnosis to operation, there were two groups of patients. Those who had episodes of acute intracranial events (SAH, ICH, SDH, hydrocephalus, acute visual problems) and those with chronic lesions (detected previously) were referred to the university hospital.

Amongst them those who had a Karnofsky performance score > 70, favorable pre-operative Hunt and Hess (HH) and Fischer grades (FG), and angiographic findings suggesting microsurgically operable lesions were selected to be scheduled for microsurgical clipping of OAA. In general, we include all the OAA in MS approach unless there are contraindications for craniotomy such as severe brain edema, anesthesia issues and other general contraindications for open occlusions.

The patients who refused to be operated on or deteriorated in medical condition were excluded from the study and treated according to their medico-legal status. Informed medical consent was obtained from all the patients and their first relatives/guardians.

### Preoperative preparation

All the patients had a recent brain CT angiography and or 4 vessel angiography and/or MRA. All of them had received the same medical regimen before the operation. Depending on aneurysm size, we defined 5 size category as maximum aneurysm diameter(MAD) < 3 mm as blister,3 < MAD < 5 as small,5 < MAD < 15 as medium,15 < MAD < 25 mm as large, and MAD > 25 mm as giant aneurysm [3].

The main pre-operative medical treatment included Nimodipine 60 mg PO every 6 h, at least 3literes intravascular N/S(NaCl 0.09%) with or without 1 L Voluven® solution, maintaining 110 < SBP < 150 mm Hg and MAP > 70 mm Hg, prophylactic intermittent pneumatic compression device(IPC) for deep venous thrombosis(DVT) prophylaxis, complete bed rest, and neurointensive care unit(NICU) care on admission. The comatose patients with acute hydrocephalus underwent external ventricular catheter placement (EVD) insertion before OAA microsurgery otherwise EVD was not a common practice.

## Microsurgery

### Position and exposure

In our center, Lateral Supra Orbital (LSO) craniotomy, as described by Hernesniemi et al. was the preferred surgical approach for most anterior circulation aneurysms even ophthalmic aneurysms [5].

**Fig. 1:**
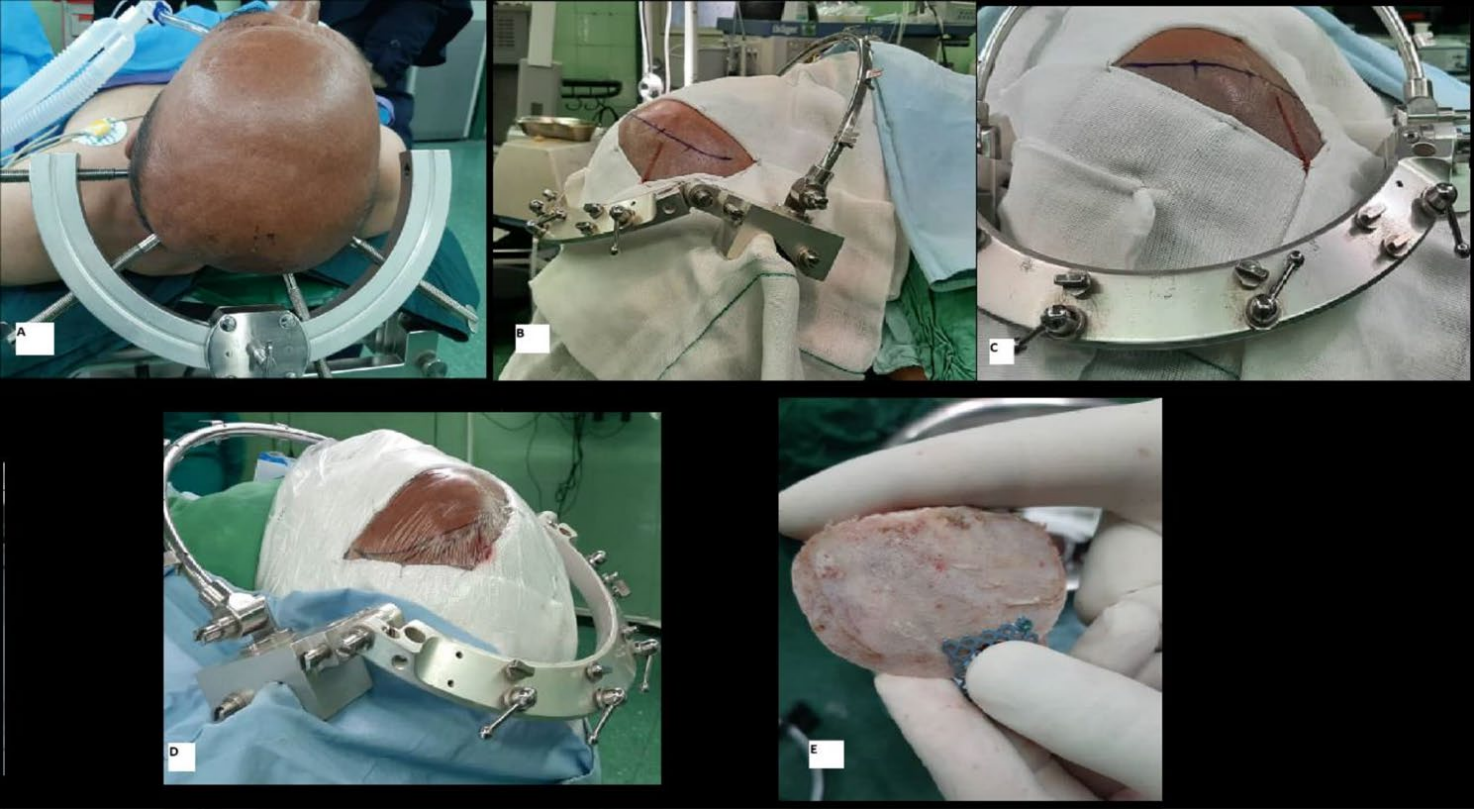
Positioning, incision, and craniotomy in lateral supraorbital approach for ophthalmic aneurysm surgery. **A**: a slight head rotation in the supine position contralateral to the aneurysm side is held. **B** and **C**: lateral and superior view of skin incisional line. **D**: the skin is draped with sterile gauze. **E**: a typical LSO craniotomy flap

Under general anesthesia and in a supine position, the head was fixed using rigid skull tongues then the head and shoulder were elevated above the cardiac level, rotated 30 degrees to the contralateral side, and tilted slightly with some degree of flexion or extension depending on surgical preferences. Proximal aneurysms located near the skull base, usually are best visualized after slight neck flexion while distal aneurysms benefit from the extension. After prep and draping in a sterile fashion, a curvilineal frontotemporal skin incision behind the hairline was performed. preserving facial nerve branches, the myocutaneous flap was deflected anteriorly to the superior orbital rim. One extra bur hole was drilled below the posterior extension of the superior temporal line and a modified 4 × 4 cm craniotomy was performed (Fig. 1). The sphenoid ridge was drilled off using a diamond bur maximizing the surgical corridor. Dura was opened in a semilunar fashion and deflected anterolaterally.

Dissection was started in the basal frontal surface and arachnoid dissection along the optic nerve and optico- carotid triangle proceeded. Using a blunt-tipped suction cannula, CSF was released from the carotid cistern. We routinely dissect and open the lamina terminalis posterior to optic chiasma for further CSF drainage. The authors found this maneuver extremely helpful since it provides further CSF evacuation thus further brain relaxation can be achieved. On the other hand, it was assumed lamina terminalis septostomy can treat and or prevent hydrocephalus development in the postoperative period. Wide Sylvian fissure dissection was performed to minimize brain retraction.

**Fig. 2:**
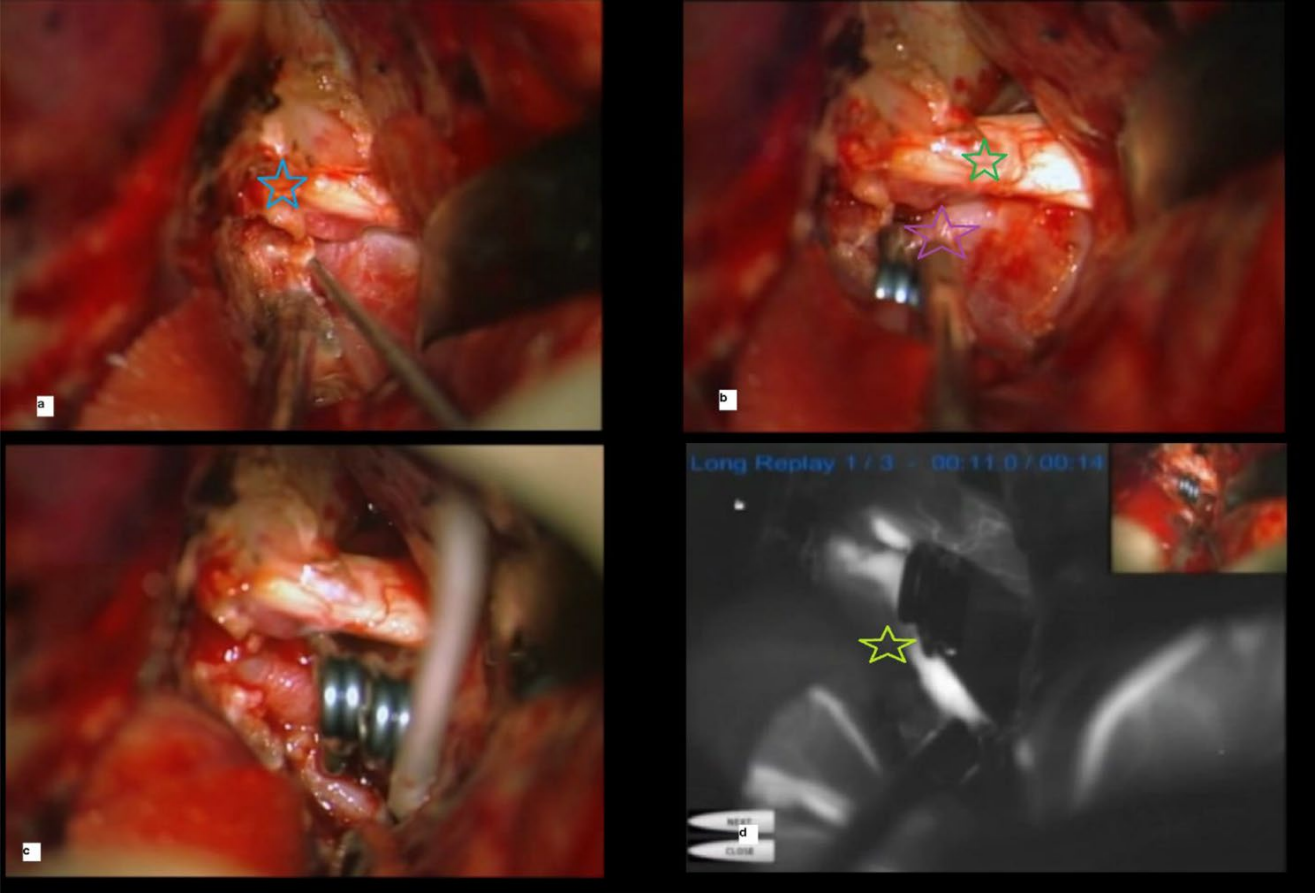
**A**: modified Intradural anterior clinoidectomy. Each stage is marked with a colored star. Blue star: anterior clinoid process residue. **B**: proximal control of clinoidal internal carotid artery, Green star: optic nerve, Purple star: aneurysm neck: tandem clip application. **D**: intraoperative fluorescein angiography is a crucial step in OAA microsurgery. The yellow star shows the parent vessel flow

Proximal control in paraclinoidal aneurysms can be achieved through the cervical internal carotid or clinoidal segment of the carotid after anterior clinoidectomy. Figure 2 represents a stepwise surgical approach for OAA obliteration. The authors used Intradural anterior clinoidectomy for proximal control simultaneously incising the falciform ligament to release the optic nerve. After clinoidectomy distal dural ring was opened and we gained access to the clinoidal segment of ICA.

### Aneurysm dissection and occlusion

Distal and proximal aneurysm necks were carefully exposed and dissected.

A proximal temporary clip was applied on the clinoidal segment then the ophthalmic artery was dissected and released from the the aneurysm complex. Under special circumstances such as challenging anatomical correlations or clinoidal ICA injuries, a cervical proximal control was predicted to be useful thus we always had the ipsilateral cervical ICA, prepped and draped.

In an ICA segment aneurysm, especially an ophthalmic aneurysm, if the aneurysm neck diameter is larger than the carotid girth, applying a standard straight or curved clip will result in carotid wall folding in multiple planes along the clip length. These wrinkles will cause carotid lumen stenosis thus predisposing the artery to thrombus formation and lumen obstruction. This phenomenon is named the *Accordion effect*. To prevent it, the authors tried a combined clip reconstruction technique to increase the occlusion rate while preserving ICA blood flow. To achieve these targets, instead of a straight or curved clip, we used fenestrated clips for OAA neck occlusion.

Fenestrated clips could possibly be associated with neck remnants on the application site beneath the fenestrated portion.to prevent any residues we applied a small straight clip on aneurysm residues which resulted in complete aneurysm occlusion while avoiding the accordion effect.

Intraoperative Doppler sonography and Indocyanine green (ICG) video angiography confirmed aneurysm occlusion preserving the patency of the parent vessel. In case of a large aneurysm or compressed optic nerve, we perforate and resect the aneurysm dome to decompress the optic apparatus. The surgical field was irrigated with a copious amount of warm sterile normal saline (N/S 0.09%) solution and small amounts of local papaverin were applied on exposed vessels to prevent vasospasm.

Hemostasis was obtained and the dura was closed in a water-tight fashion. Using titanium mini-plates and screws the bone flap was fixed in situ and the temporalis muscle was repaired using 2/0 Vicryl® sutures. A subgaleal drain was inserted and fixed, and soft tissues and skin were repaired in anatomic layers.

### Postoperative period

**Fig. 3:**
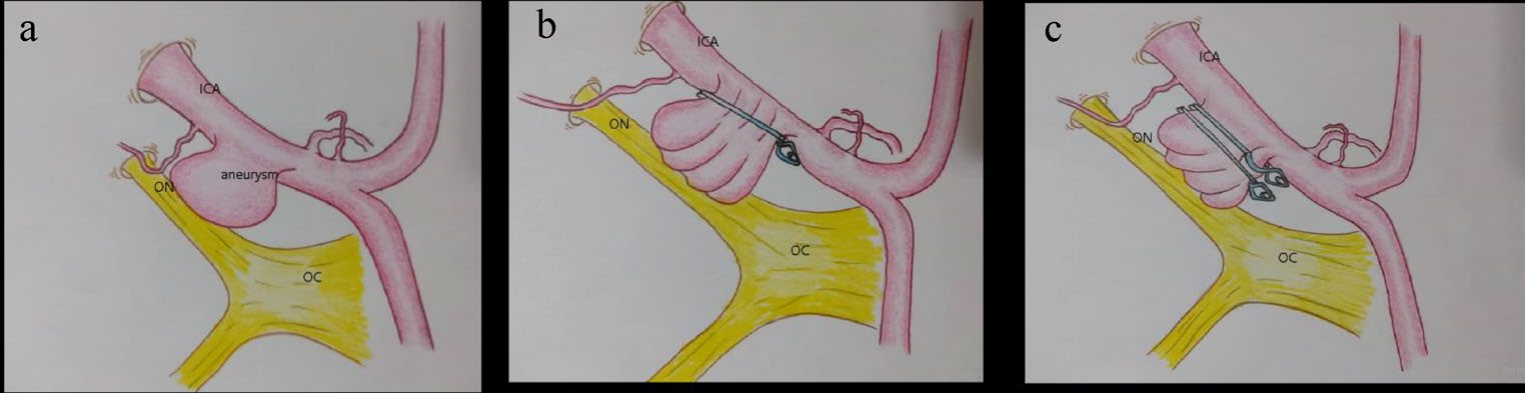
aneurysm reconstruction technique to avoid the accordion effect. **a**: internal carotid artery (ICA) aneurysm projecting on the optic nerve (ON) and chiasma(OC), **b**: single clip causes wrinkling of the aneurysm and ICA internal wall, casing accordion effect, **c**: multiple clip reconstruction yields better vessel-aneurysm wall reconstruction, accordion effect avoidance, and favorable outcomes

All the patients were transferred to the neurosurgical intensive care unit (NICU) and received routine aneurysm microsurgery care. A routine postoperative brain CT scan was taken immediately after surgery. Next brain CT scan was obtained 6 h after the initial scan. A routine CT angiography was performed on the next day to check the occlusion percent (Fig. 3). If there were considerable residues or clip malposition, the patient would be transferred to the operation room for revision surgery.

Nimodipine 60 mg PO every 6 h, phenytoin 100 mg IV every 8 h, or levetiracetam 500 mg IV every 12 h were prescribed as the core post-op orders for non-epileptic patients for 1 week and discontinued if the patient had no epileptic attacks. Post operative antibiotic regimen was Ceftazidime 1gr IV every 8 h for 3 days in combination with Vancomycin 1gr IV every 12 h for 1 day with proper renal adjustments. The drain was removed 2–5 days after operation. In cases of CSF leakage, we kept the drain for up to 5 days and the antibiotics were continued until drain removal. If the patient showed the symptoms of raised intracranial pressure, a lumbar puncture(LP) or direct ventricular pressure measurement (via EVD) was performed. If LP opening pressure/ICP > 20 cm H2O, we tried to drain 20–40 cc CSF and keep 12 < ICP < 25 cm H2O. The patients who remained dependent on EVD and had persistently raised intracranial pressure(ICP > 25 cmH2O) underwent a ventriculoperitoneal shunt procedure(VPS) to control the ICP in the normal physiologic range. Prophylactic enoxaparin 40 mg SC daily or heparin 5000 IU SC every 12 h was administered in combination with IPC unless there were contraindications of chemoprophylaxis.

If the patient showed clinical symptoms of vasospasm, hypervolemic-hypertensive-hemodilution (HHH) therapy was advised. If vasospasm was not amenable to HHH therapy, endovascular treatments were considered to alleviate vasospasm.

In terms of postoperative hemorrhagic complications depending on neurological status and amount of bleeding the patients were observed or re-operated.

The patients with poor neurological status were introduced to palliative teams for early tracheostomy and home care preparations.

### Follow up

All of the patients were appointed in university hospital clinics 2 weeks after discharge. A routine CT angiography/4 vessel angiography was obtained 6 months after the operation to investigate the durable occlusion, residual lesions, or treatment failures.

### Statistical methodology

Demographic data, physical examination findings, lab results, GCS/mRS/HH scores, in-hospital and follow-up mortality rates, new medical events, presence of emergence of hydrocephalus, hydrocephalus treatments, aneurysm anatomic characteristics and concurrent intracranial lesions, brain CT scan findings, pre and postoperative angiographic results, surgical important notes, temporary clip application duration, aneurysm clip specifications, number of clips, adjuvant endovascular/microvascular techniques, operation duration, blood loss/received, intraoperative events, ICU stay duration, hospitalization period, mechanical ventilation days, type and dosage or medications received, long terms neurological outcomes(mRS, GOS, GCS, HH, FG) and examination, adverse drug reactions, long terms(> 6 months) obliteration rate, possible treatment failure/retreats, another brain surgery for any reason, and all recordable medical findings were documented by surgical team. Favorable neurological outcomes were defined as GOS = 5 and/or mRS = 0–1 and poor neurological outcome was determined as GOS = 1–3 and/ or mRS = 4–6.

Descriptive statistics including mean ± SD and frequencies were determined for continuous and discrete variables, respectively. Normal distribution of the data was determined with The Kolmogorov-Smirnov test. Chi-square and independent t-tests were used to investigate the relations between the variables in the two groups. Pearson correlation coefficients were calculated for the relationship between eligible variables. P-value < 0.05 was considered statistically significant for all tests. Univariate and multivariate analyses were performed for correlation assessment between outcomes and predictors. Statistical analyses were conducted using IBM SPSS software (Version 23, Armonk, NY: IBM Corp.)

## Results

Amongst the 55 patients with OAA, 17 were males (30.9%) and 38 were females (69.1%). The mean value for age was 52.7 ± 1.16 years. Median values for preoperative GCS, HH, and FG were 15, 1, and 1, respectively. 32 out of 55 patients were admitted due to acute neurological symptoms and underwent angiography while 23 were referred from other centers (Table 1).In terms of pre-operative neurologic deficits(ND), 19 patients ( 34.5%) had one or more acute focal lesion(s). The most common neurologic manifestation was visual problems (*n* = 15). In terms of new postoperative visual problems, there was only 1 new oculomotor nerve palsy which was resolved conservatively.

**Table 1:** Mean values for perioperative variables, quantitative values

		Hemorrhage-to-operation interval (days)	Age(years)	GCS	Pre-op HH score	Pre-op Fisher grade	Aneurysm size	Aneurysm number	Temporary clip duration(minutes)	Intraoperative bleeding	Operation duration	GOS follow up	mRS follow up
N	Valid	32	55	55	55	55	55	55	53	55	55	55	55
	Missing	None (*n* = 23 was elective refers)	0	0	0	0	0	0	2	0	0	0	0
Mean		2.28	52.96	14.054	1.800	1.85	2.527	1.23	3.98	460.00	87.854	4.654	0.654
Median		2.00	54.00	15.000	1.00	1.00	2.00	1.00	3.00	300.00	80.00	5.00	0.00
Std. Deviation		1.905	1.166	2.2145	2.13	1.007	1.033	0.607	3.433	471.05	39.644	1.057	1.601
Minimum		0	26.00	6.00	1.00	1.00	0.00	1.00	0.00	100.00	22.00	1.00	0.00
Maximum		5	80.00	15.00	15.00	4.00	4.00	3.00	21.00	2700.00	200.00	5.00	6.00

In terms of past medical history (diabetes mellitus, hypertension, ischemic heart diseases), HTN was the most common pre-operative comorbid (60%), while DM and IHD had a lower prevalence (Table 2,14.5%, and 12.7%, respectively). 29.1% of cases had smoking habits while 18.2% had a positive history of opioid addiction. Amongst emergency department admissions (*n* = 32), the mean duration from SAH event-to-operation was 2.28 ± 1.9 days. There were 1 pre and no post-operative epileptic events in the medical records of the patients(1.8% and 0.0%, respectively).

**Table 2:** Mean values for perioperative variables, and qualitative variables

Variable	Frequency	Valid percent (%)
Male	17	30.9
Female	38	69.1
Ischemic heart disease	7	12.7
Diabetes mellitus	8	14.5
hypertension	33	60
Cigarette smoking habit	16	29.1
Opium addiction	10	18.2
Pre-operative neurological deficits	19	34.5
pre-operative hydrocephalus	17	31.9
VPS dependency	2	3.6
Preoperative seizure	1	1.8
Postoperative seizure	0	0
Intraoperative aneurysm rupture	21	38
Alive patients	50	90.9
Mortality < 6 months of surgery	5	9.1

Tables 3 and 4 summarize the aneurysm-associated variables analysis.54 patients were operated on via lateral supra-orbital (LSO) approach while only 1 case was operated on with a Pterional approach.61.8% of the patients had left-sided aneurysmal lesion(s) while 36.4% had a right-sided lesion. In terms of 3-dimensional aneurysm orientation, the most common anatomical projections were medial (43.6%) and supra-medial (20%) oriented lesions.47 out of 55 patients ( 85.5%) only had 1 ophthalmic aneurysm while multiple aneurysmal lesions (2 and more), were reported only in 14.6% of the cases.1 patient(1.8%) had a positive family history of aneurysms in first relatives. Medium size aneurysms were the most common (*n* = 27, *p* = 49.1%, MAD = 5–15 mm) while the 2nd and the 3rd most common ones were giant aneurysms (*n* = 13, *p* = 23.6%) and large ones (*n* = 10, *p* = 18.2%).

**Table 3:** Aneurysm dome directions

		Frequency	Percent	Valid Percent (%)
direction	Posterior	2	3.6	3.8
	Superior	3	5.5	5.7
	Antero-inferior	1	1.8	1.9
	Postero-infrolateral	1	1.8	1.9
	Medial	24	43.6	45.3
	Lateral	5	9.1	9.4
	posterolateral	5	9.1	9.4
	posteromedial	1	1.8	1.9
	supramedial	11	20.0	20.8
	Total	53	96.4	100.0
Missing		2	3.6	
Total		55	100.0	

**Table 4:** Aneurysm features characteristics

	features	Valid number (%)
Aneurysm side	left	34(61.8)
	right	20(36.4)
Temporary clip application	yes	52(94.5)
	no	2(3.6)
Approach	Pterional	1(1.8)
	LSO	54(98.2)
Maximum aneurysm diameter (MAD, mm)	Blister (1–3)	2(3.6)
	Small (3–5)	3(5.5)
	Medium (6–15)	27(49.1)
	Large (16–25)	10(18.2)
	Giant (26–100)	13(23.6)
Total aneurysm numbers	one	47(85.5)
	Two or more	8(14.6)

Mean values for operation time and intra-operative bleeding volume were 87.85 ± 39.64 min and 460 ± 471.05 milliliters, respectively.

In 52 patients temporary clip for proximal control was used. in 21 patients (38.2%) we experienced intraoperative aneurysm rupture (IOAR) during surgery. Aneurysm rupture was associated with longer operation duration, more bleeding volumes, longer temporary clip time, lower OS, and poor mRS and GOS outcomes (Table 5). In two patients we had ICA rupture, total blood loss was 600 and 2700 CC, and ICA was repaired primarily using 7/0 Prolene® sutures in one while in another one it was ligated, both of them had favorable neurological outcomes.

**Table 5:** Correlation between intraoperative aneurysm rupture and clinical variables. Please note those who had confirmatory multi-variate analysis results, are coupled with in-parenthesis Odds ratios (OR) and confidence intervals (CI)

variable	*p*-value
Gender	0.768
Preoperative neurologic deficit	0.308
Hydrocephalus	**0.031**
VPS dependency	**0.071**
Epilepsy	0.428
Pre-operative seizure	n/a
Direction of aneurysm	0.258
Side of aneurysm	0.653
HTN	0.428
DM	0.966
IHD	0.785
Smoking	0.586
Opium addiction	0.896
Temporary clip application	0.269
Approach (LSO vs. Pterional)	0.428
Approach side	0.714
Over-all survival	0.044
Age(years)	0.143
Bleeding to operation interval(days)	0.383
GCS	0.446
Initial HH score	0.918
Initial Fisher grade	0.166
Aneurysm size category	**0.001(OR :4.749, CI:1.842–12.244)**
Temporary clip interval	**0.004**
Bleeding amount(cc)	**< 0.001**
Operation duration(minutes)	0.093
Overall survival (OS)	**0.044**
GOS	**0.02**
mRS	**0.012(OR :1.704, CI:0.9982.908)**

Larger aneurysm size was associated with higher rates of aneurysm rupture (*p* = 0.003). in terms of other aneurysm anatomical features (side, number, orientation, type of craniotomy, and side of approach) there was no correlation between the risk of rupture and these variables (Table 5). Pre-operative hydrocephalus was associated higher incidence of aneurysm rupture (*p* = 0.031) while VPS dependency did not correlate with IOAR (*p* = 0.071). P value < 0.05 was considered statistical significant.

In terms of preoperative hydrocephalus (HCP) severity, 38 out of 55 patients had no HCP (69.1%), while the prevalence of mild, moderate, and severe HCP was 14.5%, 10.9%, and 5.5%, respectively.

In the majority of cases (94.5%) there was no need for a ventriculoperitoneal shunting procedure (VPS). Only 2 (3.6%) of the cases required VPS to compensate for existing hydrocephalus(*n* = 2). 28.5% of the patients with visual field /acuity problems had clinical improvement in the postoperative period (*p* = 4/14).In one patient(1.8%) we had to revise the clip within 24 h after immediate postoperative CTA to obliterate the small ear-dog-shaped aneurysmal residue. Two patients had postoperative CSF leak which was treated conservatively(3.6%). Two patients had another craniotomy for non-aneurysmal lesions in the early perioperative period(ICH and brain edema, Table 6).

**Table 6:** Specific medical conditions

variables	Frequency (percent %)	Description
Primary Visual field defect	2(3.6%)	
Primary Visual acuity deficit	12(21.8%)	Ranging from mild to severe
Oculomotor palsy	2(3.65)	-1 new postoperative transient palsy -1 pre-operative, improved post operation
New postoperative visual problems	0(0%)	
Postoperative visual improvement	4(28.5%)	-The percentage is calculated based on the patients with existing visual problems, 3 upper rows(*n* = 15) -one of them had improved 3rd nerve palsy postoperation
Postoperative CSF leakage	2(3.6%)	conservative management
Reoperation	3(5.4%)	-clip revision(*n* = 1) -severe brain edema, Bicoronal decompressive craniectomy(*n* = 21) -ICH due to simultaneous AVM(*n* = 1)
Severe vasospasm	1(1.8%)	Expired, alcoholic
Major vessel injury	2(3.6%)	-Complex and partially thrombosed aneurysm, ICA rupture, repaired primarily -an old patient, ICA rupture,2700 cc bleeding ligation, good functional outcome
Non- aneurysmal simultaneous vascular malformation	1(1.8%)	Frontal Arteriovenous malformation (AVM)grade III, bled after OAA surgery, operated and AVM resected.

The patients were followed for a long-term period after discharge. The postoperative follow-up period ranged from 2 years up to 12 years with a mean period of 5 years.

Median values for follow-up GOS and mRS were 5 and 0, respectively. favorable GOS and mRS scores were associated with better primary GCS, HH, and Fisher grade scores (Table 7). Intraoperative aneurysm rupture and hydrocephalus were strongly associated with poor neurological outcomes (*p* = 0.012 and 0.003, respectively, Table 7).

**Table 7:** Association between modified Rankin scale(mRS), and overall survival (OS) and clinical variables. Please note those who had confirmatory multi-variate analysis results, are coupled with in-parenthesis Odds ratios (OR) and confidence intervals (CI)

variables	OS *P* value (OR, CI)	mRS *P* value (mRS)
Intraoperative aneurysm rupture	.**044**	**0.044(4.78,1.04–21.92)**
Age	0.686	0.520
Intracranial hemorrhage-to-surgery interval	0.560	0.426
GCS	0.125	**0.002**
Preoperative HH score	0.665	0.302
Pre-operative Fisher grade	0.083	**0.001**
Aneurysm size category	0.128	0.415
Aneurysm number(s)	0.366	0.533
Total temporary clip duration	0.676	0.408
Bleeding amount	0.375	0.245
Surgery duration	0.795	0.557
OS	-	**< 0.001**
GOS	**< 0.001**	**< 0.001**
mRS	**< 0.001**	-
Hydrocephalus	**0.021(2.834 ,1.1–7.21)**	.**003**
VPS dependency	0.653	0.743
Preoperative neurological deficits	0.793	0.437
Postoperative hydrocephalus	n/a	n/a
Aneurysm dome direction	0.829	0.543
OAA side	0.888	0.273
HTN	0.348	0.455
DM	0.723	0.678
IHD	0.617	0.546
Cigarette smoking habit	0.582	0.310
Opium addiction	0.914	0.597
Craniotomy type	0.755	0.401
Application of temporary clip	0.653	0.557
Surgery side	0.862	0.290
Postoperative epilepsy	n/a	n/a

In long-term follow up 50 patients are reported to be alive (90.9%), and 5 patients (9.1%) died within the first 6 months of SAH and for any reason. Statistical analysis showed intra-operative aneurysm rupture and hydrocephalus were associated with lower OS rates (*p* = 0.044, and 0.021). P value < 0.05 was considered statistical significant.

### Case

**Fig. 4:**
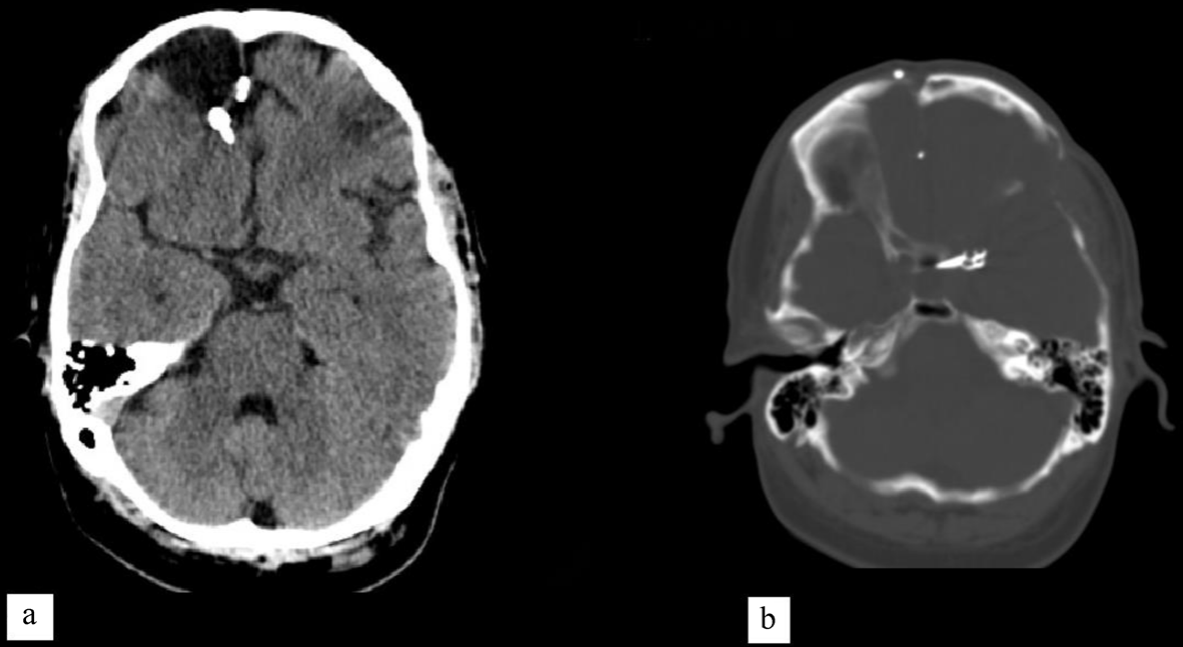
concurrent AVM and extranidal OAA in a young man who experienced hemorrhagic AVM transformation after aneurysm occlusion

An adult patient with GCS of 15, HH, and Fisher score of 1, was referred with a small left-sided supraramedially projected OAA and had an un-ruptured right frontal lobe Spetzler-Martin grade III AVM with 4 cm diameter vascular nidus, contralateral to the aneurysm. The AVM was incidentally found during angiography and was scheduled for a future operation after OAA surgery in the following weeks. OAA was secured using a microsurgical approach, bleeding volume, operation duration, and other medical conditions were in an expected range. Unfortunately, the patient experienced an unexpected ICH within 24-hour post operation in his frontal AVM and the surgical team had to perform a craniotomy and resect the AVM, long term outcome was favorable. There were no postoperative hydrocephalus, vasospasm, or epilepsy (Fig. 4).

## Discussion

Paraclinoidal zone surgery especially anterior clinoidectomy requires specific surgical expertise to preserve neural and vascular structures. post operation visual loss is an unpleasant complication of anterior clinoidectomy which is being performed in a microsurgical approach [4]. Ophthalmic artery aneurysm microsurgical obliteration in the hands of an experienced neurosurgeon and well-equipped facility will result in excellent outcomes [3].

Cerebral vasospasm is a pathological condition that most occurs between days 3 to 14 after a ruptured intracranial aneurysm. vasospasm causes cerebral hypoperfusion and/or ischemic sequela in follow. Hunt and Hess’s (HH) grading system was developed to describe the severity of clinical condition in ruptured intracranial aneurysm and predicts the clinical outcomes of the patients. Fisher and modified Fisher grading are also available to predict the clinical outcomes of aneurysms. They can reliably predict the possibility of cerebral vasospasm in the upcoming days after aneurysm rupture. All of these grading systems have acceptable reliability, validity, and reproducibility [6, 7].

In OAA treatment the approach of choice (EV vs. microsurgery) is still controversial. EVs are less invasive but have higher recurrence or retreat rates. Endovascular approaches cannot decompress surrounding neural structures and if the vessel raptures during the endovascular approach, the patients will be at great risk for severe morbidities and mortality [1, 2, 8].

In the review of the EV-focused literature, the recurrence and need for retreatment of OAA are reported frequently. *Durst et al.* reported that acceptable treatment results following pipeline embolization device (PED) application and coil embolization, were seen only in 74% and 45% of the patients( *n* = 19), respectively. They reported 11–24% retreatment intervention rates [2]. *Heran et al.*. reported 50% incomplete occlusion and 50% retreatment for OAA after the primary EV approach [9].In a multi-center study, *Adeeb* and colleagues reported 20–20% treatment failure rates in OAA endovascular treatment [10]. In 2022, Wang and Yu conducted a systematic review of EVT options for para-ophthalmic artery aneurysms. They have reviewed multiple EVT techniques such as coil embolization, FDSs, covered stents, and Woven EndoBridge devices. They concluded coiling has multiple relative superiority to FDS and, thus should be selected as the first treatment of choice in the treatment of para ophthalmic aneurysms [11].

In the review of the microsurgical-focused treatment of OAA, *Kamide et al.* reported a 91% success rate in complete occlusion of OAA and 0% retreatment in 208 of OAA following the MS approach.in our study complete occlusion of ophthalmic artery aneurysm was achieved in 100% of the patients, treatment failure was 0% and recurrence of aneurysm was 0% in long-term follow-up. *Lihara* conducted a study on 111 paraclinoidal aneurysms treated with EV and/or MS approaches. He recommended that the EV approach has lower success rates in comparison to MS approaches. On the other hand, retinal emboli are a serious complication in EV for OAA treatment. They recommended MS for paraclinoidal aneurysms especially OAA [12].

In 2017, Delgado et al. conducted a systematic review with a meta-analysis on the superiority of MS versus EVT. They concluded that none of MS or EVT had significant priority on each other, however, they declared their results had limited generalizability due to the low number and poor quality status of their data sets. They recommended larger comparative studies are required for better clinical judgment [13].

In a more recent study, Wang et al. conducted a systematic review. The authors recommended MS over EVT, especially in the young with visual deficits [11].

Visual deficits play an important role in the functional outcome of the patients. OAA is associated with visual field deficits, loss of visual acuity, or cranial nerve dysfunctions. Heran et al. retrospectively reviewed 17 patients with treated OAA. He reported vision improvement in 8 patients (50%), stabilization in 4 (25%), and deterioration in 4 (25%) [9]. Xu et al. reported almost the same results as are MS approach in paraclinoidal aneurysm surgery results [14]. Puffer reported one-fourth of OAA will develop a proximal thrombosis and this may deteriorate the visual outcomes of the patients [15]. These findings were confirmed by recent systematic reviews, as well [13].

In the current series, the prevalence of preoperative visual problems was 27.2%(*n* = 15). In terms of visual acuity/visual field defects, 28.5% (4/14) experienced clinical improvement after microsurgery. These values reflect how significantly microsurgery can improve the quality of life and visual problems of patients with OAA. We had 1.8%(*n* = 1) of new postoperative visual deterioration which was due to transient oculomotor nerve palsy. Maximum brain relaxation, almost no-touch strategy for optic apparatus, exact identification of arterial perforators/ branches during dissection and clip application, using accordion technique in aneurysm obliteration, maximum neural decompression via aneurysm dome resection and hematoma evacuation, and last but not least, surgical team vigilance are of the most leading determinants affecting visual outcomes after OAA microsurgery.

In paraclinoidal aneurysm surgery, proximal control is of paramount importance. Clinoidal and cervical segments of the internal carotid artery are options for proximal control. In this series, after proper skull base drilling and exposure, following a partial Intradural anterior clinoidectomy, we used the C5 segment(clinoidal ICA) for proximal control but always had the cervical segment of ICA(C1) prepared and draped in case of ICA rupture/damage. This precaution was extremely useful in two patients with intra-operative ICA damage. Following such a dreadful complication, induced hypotension, invasive blood product transfusion, massive intravenous fluid boluses, digital compression on ICA/common carotid, fast neck dissection to clamp carotid vessels, and carotid body manipulation avoidance are extremely helpful intraoperative measures that can save the patient’s life and reduce the morbidities.

Intraoperative electroencephalogram (EEG) and somatosensory evoked potential (SSEP) monitoring are useful intra-operative measurements that could inform the surgical team during operation about ongoing neurological ischemic sequelae. Intraoperative microvascular Doppler ultrasonography (MDU) is another beneficial tool to check the blood flow in distal arterioles before and after the occlusion site [16, 17].In a study conducted by *Xu et al.*., the mean duration for a temporary clip on C1 was 12 min(range:5–55 min). They monitored the patients’ new neurological symptoms during temporary clip on C1 with EEG, SSEP, and MDU which had desirable results [14]. In the current series, due to financial and medical supply limitations neuromonitoring was not used for any of the patients yet the postoperative outcomes remained favorable.

Better pre-operative neurological scales (HH, FG, and GCS) scales are correlated with favorable postoperative outcomes. In a single-center experience, *Radoi et al.* conducted a study on paraclinoidal aneurysm surgery outcomes. They reported best postoperative results were seen in the patients with grade 1 and 2 HH scores [18]. In our study, preoperative neurological status (defined by HH, GCS, and FG scores) was strongly correlated with long-term functional outcomes.

Long-term neurological outcomes after OAA treatment are almost favorable. Lu et al. reviewed 63 cases of OAA retrospectively. They found 85.7% favorable outcomes(GOS 4–5) in the postoperation period. *Xu et al.* reported 84% and 90% favorable GOS and mRS results after 51 large-giant paraclinoidal aneurysm surgery respectively [14].In our series, the overall survival rate was 90.9%. Good functional outcome was defined as GOS = 5 / mRS = 0–1, correspondingly 87.3% of the patients had favorable neurologic and functional status which are promising.

Post occlusion mortality rate after OAA treatment ranges between 0.5–6% [9, 14, 19]. Postoperative complications in aneurysm surgery diversify based on timing and type of event. Surgical site infection, development of new neurological deficits, worsening of existing neurological problems, postoperative hematoma, vasospasm, brain edema, infarction, hydrocephalus, ischemia, electrolytes and hormonal disturbances CSF leakage, recurrence, cardiopulmonary problems, gastrointestinal issues, venous thromboembolism(VTE) and drug side effects are among the most common postoperative medical issues after aneurysm surgery [17]. Considering paraclinoidal aneurysms and focusing on ophthalmic artery aneurysms, CSF leakage, and visual and hormonal problems can be expected as well.

Iatrogenic CSF leaks after skull base surgeries are common but avoidable. During anterior clinoidectomy, meticulous drilling of ACP, avoidance of excessive drilling in bony structures around ethmoidal air cells and sphenoid sinus, bony wax application on exposed air cells, reconstruction of skull base defects with split bone grafts, fascia, fibrin glue, and other bio-compatible materials and finally lumbar drainage insertion are the mainstay of CSF leak treatment.in most case series there are low incidence of CSF leakage after the MS approach for OAA and almost all of them were treated with the mentioned measurements [14, 16, 20].In the current series, we had 2 cases of postoperation CSF leakage (3.6%)which were treated with conservative management(Table 7). The reasons for such limited post-operative CSF leakage are multifactorial but modified anterior clinoidectomy, meticulous drilling of skull base bony structures, generous skull base augmentations after air cell breach(muscle patching, pericranium/split bone grafting and bone waxing), intra-cranial CSF diversion through lamina terminalis septostomy and surgical expertise can prevent post-surgical CSF leakage. We had no recurrence of treatment failure or surgical site infection.

In terms of concurrent AVM and aneurysm, aneurysm surgery especially in hemorrhagic lesions has priority in most of the cases, however “aneurysm first” strategy is controversial [21–23]. As discussed in the case presentation we had one patient who suffered from an intracerebral hemorrhage in his untreated AVM after OAA surgery. This event emphasizes that even though it is recommended that extranidal aneurysmal lesion and simultaneous stable AVMs, the priority is on aneurysm treatment and the AVM should be operated after aneurysm securing but this is not a flawless rule and does not guarantee that AVM will not bleed, thus the surgeons should be ready for every medical situation. Routine postoperative CT scans within postoperative days can help surgeons detect early hemorrhagic transformation in such cases.

### Limitations

Due to financial issues, we could not provide neuromonitoring for our OAA surgeries which prohibited us from its potential advantages. An intraoperative digital subtraction angiography unit could help the surgeons for better anatomical understanding during the operation but due to limited medical and financial supplies, we could not afford it. The study would have a better methodological design if it was coupled with endovascular-treated OAA cases. We had a limited number of patients in our series and this may limit the generalizability of our result, so the authors would like to recommend larger study populations coupled with endovascular-treated patients to investigate the real cons and pros of each technique in a controlled study.

## Conclusion

Microsurgical treatment of ophthalmic artery aneurysm is effective and is associated with a low risk of recurrence. Direct decompression of microsurgery is associated with improved visual outcomes.

### Electronic supplementary material

Below is the link to the electronic supplementary material.

Supplementary Material 1

Supplementary Material 2

## Data Availability

Data and original images in the current study are available from the corresponding author upon reasonable request. Authors can confirm that all relevant data are included in the article and/or its supplementary information files.

## Abbreviations

ACP: anterior clinoid process
AVM: arteriovenous malformation
CSF: cerebrospinal fluid
EV: endovascular
FG: Fischer grade
GCS: Glasgow coma scale
HH: Hunt and Hess grade
ICA: internal carotid artery
MS: Microsurgery
OAA: ophthalmic artery aneurysm
