# Supplementary material for: Microsurgical treatment of ophthalmic artery aneurysm, a case series of 55 patients with long-term follow-up
Source: BMC Surg. 2024 May 7;24:139. doi: 10.1186/s12893-024-02419-x (PMC11075342; doi:10.1186/s12893-024-02419-x)
Supplement: Supplementary file 1 — Supplementary Material 1 [file 12893_2024_2419_MOESM1_ESM.docx]

- We have conducted a case series on 55 patients with ophthalmic aneurysm microsurgical treatment and followed the outcomes.
- Visual problems improvement and complete aneurysm occlusion were resulted in 28% and 100% of the cases.
- Intraoperative aneurysm rupture was associated with preoperative hydrocephalus and larger aneurysm size.
